# Perceived social support as a factor in mental health during the COVID-19 pandemic

**DOI:** 10.1192/j.eurpsy.2022.1254

**Published:** 2022-09-01

**Authors:** L. Shaigerova, A. Dolgikh, O. Almazova, O. Vakhantseva, R. Shilko

**Affiliations:** Lomonosov Moscow State University, Psychology, Moscow, Russian Federation

**Keywords:** perceived social support, Covid-19, mental health

## Abstract

**Introduction:**

Support from a different sources can have a critical impact on a person’s mental health in a stressful situation. In situations of prolonged stress, it is necessary to identify the links between specific sources of support and mental health.

**Objectives:**

To identify the connections between different sources of social support and mental health indicators in different periods during the COVID-19 pandemic.

**Methods:**

DASS (Lovibond, Lovibond, 1995) and MSPSS (Zimet et al., 1988) were applied. The study involved 855 people aged from 18 to 60 years (M=32.9; SD=13.88). The study was conducted online in the spring of 2020 (N=426) and in the winter of 2021 (N=429).

**Results:**

Post Hoc Scheffe revealed that perceived peer support in spring 2020 was significantly lower than in winter 2021 (p<0.05). With Pearson correlation coefficient, we tested the connections between perceived social support from family, friends, and significant others and the level of depression, anxiety, and stress. In spring 2020, all mental health indicators were associated with the perceived social support from all three sources (friends, family, and significant others). In the winter of 2021, depression levels were still associated with the perceived social support from all three sources, while the levels of anxiety and stress were associated only with perceived family support.

**Conclusions:**

Thus, the study has revealed the dynamics in dependence of mental health indicators on the perceived social support from various sources at different stages of the COVID-19 pandemic. The reported study was funded by RFBR, project number 20-04-60174.

**Disclosure:**

No significant relationships.

